# Effects of Age and Muscle Type on the Chemical Composition and Quality Characteristics of Bactrian Camel (*Camelus bactrianus*) Meat

**DOI:** 10.3390/foods11071021

**Published:** 2022-03-31

**Authors:** Rendalai Si, Qin Na, Dandan Wu, Xiaoyun Wu, Liang Ming, Rimutu Ji

**Affiliations:** 1 Key Laboratory of Dairy Biotechnology and Bioengineering, Ministry of Education, College of Food Science and Engineering, Inner Mongolia Agricultural University, Hohhot 010018, China; sirendalai_imau@163.com (R.S.); naqin_imau@163.com (Q.N.); wudandan1113@163.com (D.W.); wuxy_imau@163.com (X.W.); bmlimau@163.com (L.M.); 2 Inner Mongolia Institute of Camel Research, Alxa 737300, China

**Keywords:** Bactrian camel, age, muscle type, meat quality, chemical composition

## Abstract

Camel meat could have health benefits for human consumers due to its nutritional value. The influence of age and muscle type on the chemical composition and quality characteristics of Bactrian camel meat was examined in the present study. Samples of the *Longissimus thoracic* (LT), *Semitendinosus* (ST), and *Psoas major* (PM) muscles were collected from a total of fifteen male camels in three different age groups (3–4 years, 6–7 years, and 9–10 years). The younger camels exhibited higher values of moisture, polyunsaturated fatty acids, ultimate pH, cooking loss, and lightness, but lower fat, shear force, and redness values compared to meat collected from older camels. The LT muscle had higher fat and color parameters (lightness, redness, yellowness) but lower shear force values than the ST and PM muscles (*p* < 0.05). The ST muscles had a higher content of n-6 polyunsaturated fatty acids and n-3 polyunsaturated fatty acids but lower cooking loss values than the LT and PM muscles. These results indicated that younger camels provide better meat quality traits than older camels. The results of the present study will improve the marketing of Bactrian camel meat products and will provide more information about the most suitable muscles and the optimal slaughter age.

## 1. Introduction

The Bactrian, or the two-humped, camel (*Camelus bactrianus*) is widely distributed in the arid and semi-arid areas of East and Central Asia, including China, Mongolia, Russia, and Kazakhstan, while the dromedary, or one-humped, camel (*Camelus dromedarius*) is common in the desert and semi-desert regions of Africa, the Middle East, and South Asia [1,2]. Compared to other domestic animals, camels have a unique ability to survive and reproduce in harsh climatic conditions, including in high temperature, low rainfall, and food scarcity conditions [3,4]. China is one of the main distribution areas of Bactrian camels worldwide. There are 405,300 camels in China, 42.72% (173,000) of which are found in the Gobi Desert areas of Inner Mongolia [5], and all of them are Bactrian camels [6]. In China, Bactrian camels have multiple uses, such as for meat, milk, and wool production; transportation; and value-added products. There have been many studies on the chemical composition and quality characteristics of dromedary camel meat; however, there has been limited research on Bactrian camel meat and on the development of camel meat products in China in particular.

Camel meat is considered to be suitable for human consumption because of its high protein, low fat, and low cholesterol characteristics [7]. Moreover, it is a good source of minerals, essential amino acids, vitamins, biologically active compounds, and essential fatty acids, such as n-6/n-3 fatty acids [8]. Camel meat contains 76–78% water, 19–22% protein, 2.9–3% fat, and 1.2% ash; therefore, compared to other meat-producing animals, such as cattle and sheep, camel meat is particularly lean and is thus more suitable for the nutritional demands of consumers [9]. In addition, camel meat is traditionally used to remedy various diseases, such as sciatica, hyperacidity, cardiovascular disease, hypertension, pneumonia, and respiratory diseases, and it has also been claimed to have aphrodisiac properties [10].

The chemical composition and quality characteristics of camel meat vary according to age, sex, muscle type, breed type, feeding conditions, and site on the carcass [11]. Conventionally, camel meat is produced from older animals (more than 7 years old), who have been employed in transportation, racing, milk production, or other functions [10]. Therefore, consumers generally consider camel meat to be tough, coarse, and firm [10]. Another study noted that younger dromedary camel meat has acceptable meat quality characteristics [12]. Similarly, previous research concluded that dromedary camel meat of different ages has different compositions and meat quality characteristics, which should be considered when slaughtering camels for meat consumption [7,12].

In addition to age, muscle type is another important factor that affects the quality of camel meat. Moreover, shear force, color, nutrient composition, and other indicators often differ for different parts of the animal.

The *Longissimus thoracic* (LT), *Semitendinosus* (ST), and *Psoas major* (PM) muscles are of special importance to meat producers and consumers. The LT is the most commonly studied muscle in meat research. Its fat content is higher than that of the ST and PM muscles, but its moisture and protein contents are lower. According to previous research, the chemical composition of the ST muscle can be similar to that of the PM muscle [11,13]. For these reasons, the present study investigated these three muscles from nutritional, quality, and economic points of view.

The study aimed to investigate the effects of age and muscle type on the chemical composition and meat characteristics of Bactrian camel meat and to provide a theoretical basis for improving the marketing of camel meat products and to further provide more information about the most suitable muscles at the optimal slaughter age.

## 2. Materials and Methods

### 2.1. Animals and Meat Samples

The geographical coordinates of the study area were from 99°44′ to 104°38′ E and from 38°38′ to 42°02′ N in a pasture in Alxa League, Inner Mongolia, China. This area part of a warm-temperate desert and arid area and has a typical dry continental climate. It has four distinct seasons. The average annual temperature is 8.4 °C, the average temperature in January is −7.8 °C, and the average temperature in July is 25.4 °C. The annual average precipitation is 113 mm, whereas the annual sunshine hours are 3104.6 h, and on average, 78 days experience gales. Therefore, it is very suitable for camel survival: a total of 37% of Chinese Bactrian camels are distributed in this area [5].

In this study, fifteen male Alxa Bactrian camels purchased from local producers were classified into three age groups based on body weight: age group 1 (3–4 years old, *n* = 5, body weight 370 ± 8.7 kg), age group 2 (6–7 years old, *n* = 5, body weight 705 ± 9.4 kg), and age group 3 (9–10 years old, *n* = 5, body weight 973 ± 11.9 kg). Based on the research guidelines of the Inner Mongolia agricultural university, all animals were reared in pastoral areas and only grazed on natural grasses in Alxa League, China. All of them were kept under the same environmental conditions and were under the same management conditions for 3 months to attain a similar nutritional background before slaughter. The animal study was reviewed and approved by the Animal Administration and Ethics Committee of the Camel Protection Association of Inner Mongolia (Permit No. 2020-008).

The experimental camels were starved for 12 h before slaughter, and water was available at all times. They were slaughtered at the Lifa Agriculture and Animal Husbandry Industry Co., Ltd., Inner Mongolia, China. Slaughterhouse in Alxa League. The animals were slaughtered according to the food industry-approved Halal food quality certified protocols guided by the tenets of Islam [14]. The ambient temperature at the slaughterhouse ranged between 25 and 27 °C. In this slaughter procedure [7], the camels were bled without stunning. After exsanguination, the carcasses were split into two equal halves along the vertebrate column, and then the three different muscles, *Longissimus thoracic* (LT, at the 12th–13th rib), *Semitendinosus* (ST, at the thickest part of the hind leg), and *Psoas major* (PM, at the central portion), were collected from the left side of the 15 camel carcasses within 20 min post-slaughter. Samples were kept in zipped plastic bags (polypropylene) that had been purchased from a local supermarket and were transported in an insulated cool box (4 °C) from the slaughterhouse to the meat laboratory at the Inner Mongolia Agricultural University. A total of 6 h after arriving at the laboratory, all samples were taken out of the plastic bags, and each muscle was trimmed of external fat and connective tissue, washed with chilled deionized water, and kept in a chiller (3–4 °C) for 48 h [3,11,12]. Thereafter, the samples were vacuum packaged (Packaging Sealing Machine, QT-124, China) in polyvinylidene chloride bags (Zhenzhun Biotechnology Co., Ltd., Shanghai, China; pore size 8 cm, thickness 70 μm, O_2_ diffusion rates 0.05 cm^3^·m^2^·d^−1^) and stored at −20 °C for further chemical composition and quality measurements.

### 2.2. Chemical Analysis

The proximate chemical composition of the muscle tissue was determined according to the procedure described by Liu et al. [15], and the samples to be tested were thawed at 4 °C for 24 h before testing and were analyzed in the same laboratory. Moisture was measured using the direct drying method (at 105 °C), protein was tested using the Kjeldahl method, fat was determined using the Soxhlet extraction method, and the ash content was determined via ashing samples in a muffle furnace at 500 °C for 24 h, respectively. The mineral levels (phosphorus, calcium, magnesium, sodium, potassium, zinc, iron, lead, selenium, and copper) were evaluated following the Chinese recommended standard (GB5009.268-2016) using previously described methods (ICP, Inductively Coupled Plasma) [16] with some modifications.

Amino acids were quantified based on the Chinese recommended standards (GB 5009 124-2016) described by Liu et al. and Dai et al. [15,17], with some modifications. The protein was broken up into free amino acids by acid hydrolysis at 105 °C for 22 h and then separated using an AJS-01 amino acid special analytical column (C18, 3 μm, 4.6 × 150 mm; Welch Technology Co., Ltd., Shanghai, China) and derivatized using a ninhydrin solution. The derivatives were detected using a high-performance liquid chromatography system (HPLC, LC-20A, Shimadzu; fluorescence detector, RF20A, Shimadzu, Japan). The flow rate of the eluent was 1.6 mL/min, while the injection volume was 2 µL. The absorbance wavelength was found to be 340 nm. The column temperature was controlled at 50 °C. The content of each amino acid was calculated based on the derivatives, which were determined under the above conditions. The amino acid contents are expressed as the average of three replicate tests in g/100 g, as described previously [15,17].

The fatty acids were detected based on the Chinese recommended standard (GB5009.168-2016) using previously described methods [15,18] with some modifications. The sample was extracted using a mixed solution of chloroform and methanol (*v*:*v*, 2:1) to obtain the fat. Next, each camel fat sample (10 mg) was weighed, transferred into a centrifuge tube, and hydrolyzed and decomposed into fatty acids using 2 mL of 0.1 M methanolic sodium hydroxide solution. Then, 2 mL of a boron fluoride–methanol solution was added to obtain the fatty acids. Finally, 2 mL of n-hexane was added to extract the fatty acid methyl esters. A gas chromatographic system (SCION 456-GC, STS Shanghai Analytical Instrument Co., Ltd., Shanghai, China) combined with a flame ionization detector (FID) and Agilent J&WCP-Sil88FA- ME capillary column (100 × 0.25 mm × 0.20 µm; Agilent Technology, Santa Clara, CA, USA) was used to analyze the contents of the fatty acid methyl esters. The temperatures of the injector and detector were set at 270 and 280 °C, respectively; the carrier gas was nitrogen; the split ratio was 100:1; and the injection volume was 1.0 μL. The detection conditions needed to satisfy the theoretical plate number (*n*) of at least 2000/m, and the resolution (R) was at least 1.25. The percentage of fatty acids was calculated using the area normalization method. Determinations were made on each of the collected muscle samples in triplicate.

fatty acid *Yi* (%) = (*A_Si_* × *F*_FAME*i*-FA*i*_)/∑*A_Si_* × *F*_FAME*i*-FA*i*_

where:

*Yi* represents the percentage of a certain fatty acid in the sample to the total fatty acid, %;
*A_Si_* represents the peak area of each fatty acid methyl ester in the sample measurement solution;
*F*_FAME*i*-FA*i*_ represents fatty acid methyl ester *i* conversion coefficient to fatty acid;
∑*A_Si_* represents the sum of the peak area of each fatty acid methyl ester in the sample measurement solution.

### 2.3. Meat-Quality Evaluation

Meat quality, including ultimate muscle pH, cooking loss percent, Warner–Bratzler shear force, and color values (a* for redness, L* for lightness, and b* for yellowness), was determined. Muscle samples were thawed overnight at 4 °C before the measurements took place. The Chinese recommended standard (GB5009.237-2016) method was used to determine the ultimate pH value of the camel meat as follows: The sample was diced into minced meat using a porous meat grinder (aperture: 3 mm), weighed to 10.0 g, added to 90 mL water, and homogenized (using an Ultra Turrax T25 homogenizer) for 30 min in a water bath at 22 °C, and the pH of the filtrate was determined using a pH meter equipped with a glass electrode (FE28 Benchtop pH Meter, Mettler-Toledo, Leicester, UK). The pH meter was calibrated with pH 4.01 and 6.86 buffers at 22 °C.

Color values were assessed using a chromameter (Konica Minolta, CR-400, Tokyo, Japan). The determination was based on previously described methods [19,20,21]. Approximately 40 min after exposing the fresh surface of the muscle samples, reflectance measurements were recorded. The chromameter was set to the L* (lightness), a* (redness), and b* (yellowness) color space and illuminant D65, an observer angle of 2°, and an aperture size of 5.0 mm with a closed cone. The chromameter was calibrated using a standardized white tile before measurement. Three spots were examined for each sample.

A total of 400 g of each meat sample (6 cm × 3 cm × 3 cm) was taken to measure the cooking loss value. All of the muscle samples were placed in a plastic bag and cooked in a water bath at 100 °C to an internal temperature (the center temperature of the meat) of 70 °C. The internal temperature was tested by inserting a thermocouple probe (NR81539, TES, Taiwan) into the center of each muscle sample. Then, the meat samples were cooled to room temperature (approximately 20 °C). The muscle surfaces were dried with filter paper and reweighed using an analytical balance. Cooking losses were calculated as the difference between the weight before cooking (raw weight) and the weight of the muscle samples after cooking (cooked weight), as described by Suliman et al. [13]. Three different muscles (LT, ST, and PM) from three different age groups (3–4 years, 6–7 years, and 9–10 years) were randomly selected and assigned to a cooking batch.

Cooking loss (%) = (weight of raw sample − weight of cooked sample) × 100/weight of raw sample.

The shear force of the meat samples was assessed following a previously published procedure [19,20,22]. The samples used for measuring cooking loss were reused to assess the shear force. The shear force was obtained as the maximum force (N) perpendicular to the fibers using a TA-XT ExpressC Texture Analyzer (Weixun Super Technology Co., Ltd., Beijing, China) fitted with a Warner–Bratzler attachment. The parameters were set as follows: the pre-test speed was 2 mm/s, the post-test speed was 2 mm/s, the test distance was 20 mm, and the trigger force was 5 g.

For all of the determinations, three readings were taken, and the mean value was calculated.

### 2.4. Statistical Analysis

Microsoft Excel 2010 (Microsoft Corp., Redmond, WA, USA) was used to organize the experimental data, and the results were statistically analyzed by *IBM SPSS Statistics 26.0* (IBM Corp., Armonk, NY, USA). Data from three muscle types of LT, PM, and ST from Bactrian camels in three different age groups (3–4 years, 6–7 years, and 9–10 years) were analyzed using a model with fixed effects for age, muscle type, and age × muscle type interaction in a two-way analysis of variance (ANOVA). The ANOVA tables that were obtained were further analyzed to compare the means using least significant difference (LSD) procedures. A difference was considered significant at *p* < 0.05. All the data are expressed as the mean ± standard error of the mean (SEM).

## 3. Results

### 3.1. Effects of Age and Muscle Type on Meat Chemical Composition

The means of the chemical composition of the Alxa Bactrian camel meat from different age groups are shown in Table 1. There was an effect of age and muscle type on moisture and fat (*p* < 0.05) but no effect of age × muscle type interaction (*p* ≥ 0.05).

Age had a significant effect on the moisture, fat, and protein values (*p* < 0.05), with the younger camels (age group 1) having higher moisture values than the middle-aged (age group 2) and older animals (age group 3), except for PM. Furthermore, the meat samples from older animals were higher in fat and protein than those from younger camels. However, no significant differences were observed with regard to the ash percentage among the age groups (*p* ≥ 0.05).

In addition, significant differences were detected for the moisture and fat values between muscle types. The LT muscle recorded the highest fat values (7.78%, *p* < 0.05) and had the lowest moisture content (70.89%) compared to those in the ST and PM samples from older camels. In younger camels, the LT (3.66%) muscle had 175% and 815% more fat than the PM (1.22%) muscle and ST (0.4%) muscle, respectively. This result showed that the fat content in younger camel meat is very low, especially in the ST and PM muscles. Simultaneously, the ST and PM muscle samples exhibited significant differences in terms of the moisture content compared to the LT samples (*p* < 0.05); however, there was no difference between the ST and PM samples (*p* ≥ 0.05). Similarly, there were no significant differences in terms of the protein and ash contents between the muscle types (ST, PM, and LT). The moisture: protein ratio, which indicates the processing characteristics of camel meat, was significantly affected by age in the present study.

### 3.2. Effects of Age and Muscle Type on Meat Amino Acids

The amino acid (AA) contents of the muscle samples are shown in Table 2. Age had an effect on the total amino acids (TAA), essential amino acids (EAA), and non-essential amino acids (NEAA) (*p* < 0.05); the muscle type and age × muscle type interaction showed no significant effect (*p* ≥ 0.05). Age had a significant effect on the amino acid content (*p* < 0.05), with the LT muscle samples from older camels having higher aspartic acid (Asp), alanine (Ala), valine (Val), methionine (Met), Isoleucine (Ileu), leucine (Leu), tyrosine (Tyr), phenylalanine (Phe), histidine (His), lysine (Lys), arginine (Arg), and proline (Pro) contents than the younger camels; however, no differences were observed between age group 3 and age group 2 (*p* ≥ 0.05). In addition, age also had a significant effect on the amino acid content of the ST muscle (*p* < 0.05). In age group 3, the glycine (Gly), alanine (Ala), phenylalanine (Phe), and proline (Pro) contents (0.96, 1.26, 0.87, and 0.74 g/100 g, respectively) were higher than they were in age group 1 (0.83, 1.11, 0.77, and 0.66 g/100 g, respectively). EAA are vital for human health because these chemicals cannot be synthesized in the human body and must be ingested; hence, they are called dietary essential amino acids. The EAA content of the LT and ST muscle samples differed between age group 1 and age group 3 (*p* < 0.05) (LT contains 7.20 g/100 g, 8.19 g/100 g, and ST contains 7.27 g/100 g, 8.45 g/100 g, respectively, in age group 1 and age group 3). However, no significant differences were observed in terms of the amino acid content between the ST, LT, and PM muscle samples in this study. Regardless of age group or muscle type, the EAA/TAA values and EAA/NEAA values remained higher than 0.3961 and 0.6559, respectively. This amino acid content and ratio indicates that camel meat is a source of good-quality protein [23].

### 3.3. Effects of Age and Muscle Type on Minerals in Meat

The macro- and microelements of Bactrian camel meat are shown in Table 3. Potassium (K) was the most abundant element followed by phosphorus (P), sodium (Na), magnesium (Mg), and calcium (Ca). Age had an effect on the potassium (K), phosphorus (P), iron (Fe), selenium (Se), and copper (Cu) contents (*p* < 0.05), but muscle type and age × muscle type interaction had no significant effect (*p* ≥ 0.05).

Age had a significant effect on the macro- and microelements; in particular, the K, P, and Fe contents changed with age (*p* < 0.05). The ST, PM, and LT muscle samples from age group 2 exhibited higher Fe contents than those from age group 1 and age group 3. The LT muscle from age group 2 demonstrated higher K, P, and Fe contents than those from age group 1 and age group 3 (*p* < 0.05). However, the ST muscle from age group 2 had higher P, Zn, and Fe contents than those from age group 1 and age group 3. In addition, age had a significant effect on the contents of Se and Cu in the ST, LT, or PM muscle samples from Bactrian camels (*p* < 0.05). A significant increase in the K, Zn, and Mg levels was observed in the ST muscle (422.80, 3.44, and 25.26 mg/100 g, respectively) when compared to the PM (412.75, 2.65, and 25.08 mg/100 g, respectively) and LT muscles (402.60, 3.11, and 24.12 mg/100 g), with no significant differences being noted between the latter two muscle samples (*p* ≥ 0.05). In addition, the contents of Ca (4.594 mg/100 g) and Na (55.56 mg/100 g) in the ST muscle were higher than they were in the LT (3.69 mg/100 g, 52.68 mg/100 g, respectively) and PM muscle samples (4.33 mg/100 g, 54.45 mg/100 g), although no statistically significant differences were observed.

### 3.4. Effects of Age and Muscle Type on Fatty Acids in Meat

The fatty acid composition, total saturated (SFA), unsaturated (UFA), monounsaturated (MUFA), and polyunsaturated (PUFA) fatty acids of Bactrian camel muscles are shown in Table 4. Each muscle type had an effect on the SFA, UFA, and MUFA contents (*p* < 0.05), but age and age × muscle type interaction showed no significant effect (*p* ≥ 0.05). In contrast, age, muscle type, and age × muscle type interaction had an effect on the PUFA, UFA/SFA, and the ratio of n-6 polyunsaturated fatty acids and n-3 polyunsaturated fatty acids (n-6/n-3) as well as on the n-6 polyunsaturated fatty acid (n-6) content (*p* < 0.05). For the n-3 PUFA (n-3) content, age and muscle type showed a significant effect (*p* < 0.05), but no effect of age × muscle type interaction could be observed (*p* ≥ 0.05).

Palmitic acid (C16:0) was the most abundant saturated fatty acid in camel the PM, LT, and ST muscle samples, followed by stearic acid (C18:0) and myristic acid (C14:0); however, their contents were affected by age. The content of C16:0 gradually increased with age. The camel muscles from age group 3 (ST, LT, and PM; 29.66, 34.03, and 32.46%, respectively) contained higher C16:0 than age group 2 (ST, LT, and PM; 28.16, 30.33, and 30.99%, respectively) and age group 1 (ST, LT, and PM; 22.22, 26.40, and 25.10%, respectively) (*p* < 0.05). Simultaneously, it can be seen that the C18:0 content decreased with age (*p* < 0.05).

The main monounsaturated fatty acids found in the three muscles were oleic acid (C18:1n9c), followed by palmitoleic acid (C16:1). However, age only exhibited a significant effect on the oleic acid (C18:1n9c) content of the ST muscle (*p* < 0.05). The ST muscle sample from age group 3 showed higher levels of C18:1n9c than from the samples from age group 1 did, show levels of 35.98% and 31.58%, respectively. Age had a significant effect on the C16:1 content in the muscles (*p* < 0.05). The C16:1 levels in the ST, LT, and PM muscles from age group 3, age group 2, and age group 1 were 4.83%, 4.11%, and 3.95%; 4.12%, 3.40%, and 3.22%; and 3.22%, 2.81%, and 2.31% g, respectively.

The most abundant polyunsaturated fatty acid in Bactrian camel muscle was linoleic acid (C18:2n6c), and it was present in the ST, LT, and PM muscle samples from age group 2 at the levels of 13.71%, 4.14%, and 7.44%, respectively. These results are in accordance with other research [24]. The C18:2n6c content in camel muscles decreased with age (*p* < 0.05), with age group 1 having higher contents than age group 2 or age group 3.

The mean total saturated fatty acids were 57.83%, 58.88%, and 49.24% for the PM, LT, and ST muscle samples, respectively, and there were significant differences among the three muscles (*p* < 0.05). Meanwhile, the LT muscle contained more total saturated fatty acids than the ST and PM muscles; however, the ST muscle contained more unsaturated fatty acids (MUSFA and PUSFA) than the PM and LT muscles. Another important finding was that age had a significant effect on the content of n-3 and n-6 in the ST, LT, and PM muscles (*p* < 0.05). When the camels were 3–4 years old, the n-6 content in the PM, LT, and ST muscles was 7.53%, 4.18%, and 13.77%, respectively. When the camels were 6–7 years old, the n-6 content in the ST, LT, and PM muscles was 3.95%, 3.03%, and 6.55%, respectively. When they were 9–10 years old, the n-6 content in the ST, LT, and PM muscles was 4.87%, 2.69%, and 6.24%, respectively. It was also found that there were significant differences between muscle types (*p* < 0.05). Regardless of age, the n-6 and n-3 contents in the ST muscle were much higher than they were in the LT or PM muscles.

### 3.5. Effects of Age and Muscle Type on Meat Quality Characteristics

The mean meat quality characteristics of the Bactrian camel are presented in Table 5, including the ultimate pH, shear force, cooking loss, and color parameter (L*, a*, b*) values.

#### 3.5.1. Effects of Age and Muscle Type on Meat Ultimate pH and Color Components

There was an effect of age, muscle type, and age × muscle type interaction on the ultimate pH value (*p* < 0.05). The LT muscle of the younger camels (age group 1) had a significantly higher ultimate pH value (6.08) than that of older animals (age group 3) (5.49). The value for middle-aged camels (age group 2) (5.75) was not significantly different from that of the younger and older camels. However, for the ST muscle, the camels from age group 2 had a significantly lower ultimate pH value (5.67) than the animals from age group 1 (6.12). However, the age group 3 value (5.84) was not significantly different from age group 1 and age group 2 values. Concerning the muscle type, the LT (6.08, 5.75) and ST (6.12, 5.67) muscles from age group 1 and age group 2, respectively, exhibited higher ultimate pH values than the PM muscle (5.55, 5.52), whereas there were no differences between the LT and ST muscles. Furthermore, the ultimate pH values of the LT, ST and PM muscles from age group 3 were not significantly different.

In addition, age and muscle type had an effect on the color parameters (L* and a*) (*p* < 0.05), no effect of age × muscle type interaction was observed (*p* ≥ 0.05). For color parameter b*, muscle type differed (*p* < 0.05), while age and the two-way age × muscle type interaction did not differ (*p* ≥ 0.05). The PM, LT, and ST muscles from the younger camels had higher L* values than those from older camels did, while the reverse trend was observed for the a* values. Regardless of the muscle type, the L* value in the muscle gradually decreased with age, while the a* value gradually increased. Age also had a significant effect on the b* value of the PM muscle, where meat from older camels demonstrated higher b* values than the meat from younger camels did (*p* < 0.05).

There were also significant differences in the color indicators between the different types of muscles from camels that were the same age (*p* < 0.05). When the camels were younger (age group 1), the L* values in the PM muscle were significantly higher than those from the ST muscle. However, in the older camels (>8 years old), the a* values in the LT muscle were significantly higher than those in the ST muscle. In addition, the b* values of the LT muscle were higher than those of the PM and ST muscles. In summary, the LT muscle had higher color values (L*, a*, and b*) than the ST and PM muscles.

#### 3.5.2. Effects on Shear Force and Cooking Loss

Meat quality attributes, including the cooking loss and shear force values, were significantly affected by age and muscle type and are presented in Table 5. Age and muscle type had an effect on the cooking loss and shear force values (*p* < 0.05), age × muscle type interaction had no effect (*p* ≥ 0.05). The most marked differences in the meat quality characteristics among the three age groups were for tenderness, which is expressed by the shear force. The shear force was higher for the older camels (age group 3) (121.27 N, 115.58 N, and 132.49 N for the PM, LT, and ST muscles, respectively) than it was for the younger animals (age group 1) (84.57 N, 80.74 N, and 93.52 N for the PM, LT, and ST muscles, respectively) (*p* < 0.05), while the middle-aged camels (age group 2) were in between (102.51 N, 93.66 N, and 112.36 N). Regarding muscle type, the LT and PM muscles had a significantly lower shear force than the ST muscles, whereas there was no difference between the first two muscles. Therefore, we can infer that the LT muscle is more tender than the other two muscles (PM and ST).

The effect of age on cooking loss is also shown in Table 5. The muscles from the younger camels had higher values than those of the older animals (*p* < 0.05). The value from the middle-aged camels was in between. Significant differences were also observed in relation to the type of muscle. The PM muscle had a higher cooking loss value than the ST muscle (*p* < 0.05), whereas no significant differences were observed between the PM and LT muscles.

## 4. Discussion

### 4.1. Effects of Age and Muscle Type on Meat Chemical Composition

The chemical composition of meat is considered to be an important indicator for the measurement of meat functionality. Protein, fat, and minerals are essential constituents that reflect meat quality, and the moisture content plays an vital role in the processing potential, shelf life, and quality maintenance of camel meat [9].

Age had a certain impact on the protein and fat content of camel meat. In the current study, the protein and fat contents of the camels from age group 3 were higher than those in age group 1 (*p* < 0.05). For the ST and LT muscles, age had a certain effect on moisture, with age group 1 having a higher moisture content than age group 3 (*p* < 0.05).

Concerning the muscle type, the present results showed higher moisture contents in the ST and PM muscles compared to in the LT muscle from age groups 2 and 3 (*p* < 0.05). In the younger camels, the moisture content in the ST muscle was higher than that in the LT and PM muscles; however, no significant differences were observed between them. In the present study, the moisture content of the LT and ST muscles (from younger camels) was 74.52% and 75.75%, respectively, and both values are higher than those (73.80%, 75.40%) from one-humped camels reported by Kadim et al. [25]. A mean moisture content of 73–78% has been reported previously [2] for camels in Saudi Arabia. These differences might result from variations in breed, sex, pre- and post-slaughtering handling, or the age of the animals.

The older camels had a higher protein content than the younger camels (*p* < 0.05). Previous studies reported that the normal protein content range in camel meat is approximately 17–23.7% [3,11,12]. In the current study, as age increased, the protein content of camel meat fluctuated in the range of 19.6–22.42%, which is in accordance with the normal range of values [7,11,25]. Compared to other animal meats, camel meat (21.83%) has a significantly higher protein content than beef (20.64%) and mutton (21.62%) [23]. This level of protein content indicates that camel meat is a good source of protein in areas with low rainfall and harsh climates. However, no significant differences were observed between the protein contents among the PM, LT, and ST muscle samples in the present study.

In the present study, the muscles from older camels (age group 3) had lower moisture:protein ratios than from the younger camels did (age group 1) (*p* < 0.05). The ranges of the moisture:protein ratio in the PM, LT, and ST samples demonstrates that camel meat is suitable for further processing [3,11,12]. The significance of the moisture:protein ratio in camel muscle is its obvious effects on its shelf-life, processing performance, and quality characteristics. Generally, consumers prefer juicy meat over dry meat [7].

The present study found that the fat content in camel meat was significantly affected by age. The muscles from older camels (>4 years, age group 2 and 3) contained significantly more fat than those from younger camels (3–4 years). The fat content range (0.4–7.78%) from the present study confirmed that camel meat could be much leaner than other types of meat, such as mutton, beef, and pork [25], especially when the camels are slaughtered at a young age. Regarding the muscle type, the fat content in the LT muscle was significantly higher than that in the ST and PM muscles, and the maximum value (7.78%) was also noted in the LT muscle in older camels (>9 years). It has been suggested that this difference might be related to the lower moisture content in the LT muscle [26]. The mean fat value of 5.21% for Bactrian camel LT muscles in this study was lower than the value (6.2%) obtained in Kadim’s study [11], but higher than the mean (4.37%) reported by Suliman [13]. As a result of the differences in the slaughter age, sex, and variety, variability in the average fat content is expected for camel meat. However, the fluctuating range of the fat content recorded in this study indicates that the fat content in Bactrian camel meat increases significantly with age. It is common to find a high fat content in meat-producing animals, and with age, fat deposits increase, i.e., fat is a late-maturing body tissue [11]. Pérez et al. (2000) reported that adult llamas (>3 year) tended to have less moisture and higher fat than younger animals (<1 year) [27]. As age increases, the fat content in yak meat from different age groups has been shown to increase significantly (*p* < 0.05). Likewise, the fat content of meat from younger yaks (<3 years) was only 1.05% and increased to 2.37% from older yaks (7 years) [28]. Thus, the meat industry should target younger camels for the production of high-quality meat.

The ash content of camel meat was not significantly affected by age. The ash values of the muscles from camels belonging to the three different ages ranged from 1.11% to 1.39%, which is consistent with the values reported by other studies [8,12]. Furthermore, no significant differences were observed in the ash content among the muscle types.

Amino acids are an important indicator for the evaluation of meat protein quality. The total amino acid content of Bactrian camel meat was higher than that of mutton [23] and close to that of yak meat [15]. This study showed that age has a significant effect on the amino acid content of the LT muscle. The LT muscles from the older camels had higher values of Asp, Ala, Val, Met, Ile, Leu, Tyr, Phe, His, Lys, Arg, and Pro than those from the younger camels. Similarly, as age increased, the TAA content and EAA contents gradually increased. This phenomenon is supported by data from the LT and ST muscles. The TAA and EAA contents of the LT and ST muscles of old Bactrian camels (age group 3) were higher than those from younger camels (age group 1) and middle-aged camels (age group 2). The EAA/TAA and EAA/NEAA values were not significantly different between the age groups or muscle types. Comparing the amino acid content in Bactrian camel meat with the ideal protein model proposed by the FAO/WHO, the EAA/TAA ratio in high-quality meat should be around 40%, and EAA/NEAA ratio should be above 60%. In this study, the value of the EAA/TAA ratio in Bactrian camel meat was 39.61–39.97%, and the value of the EAA/NEAA ratio was 65.59–66.58%, both of which are in line with the ideal protein standards proposed by the FAO/WHO.

The fatty acid content analysis showed that SFAs are the main fatty acid component in Bactrian camel meat. Compared to the ST and PM muscles, the LT muscle had the highest SFA content, in which palmitic acid and stearic acid were the most abundant. The total monounsaturated fatty acid content in camel meat was not affected by age but was significantly affected by muscle type. The ST muscle demonstrated significantly higher values than the PM and LT muscles. At a younger age, the percentage of polyunsaturated fatty acids in the camel meat (6.02–16.75%) was within the range reported for beef (8.8%), buffalo meat (28.6%), and deer meat (31.4%) [29]. The PUFA, PUFA/SFA, n-6/n-3, n-6, and n-3 values are indicators that can be used to evaluate the nutritional value of meat [15,17]. PUFAs can make meat more aromatic while cooking [3,4]. The PUFA/SFA content in beef and mutton is about 0.1, and the value recommended by nutritionists is 0.4 [30]. In this study, the PUFA/SFA value of the ST muscle of young camels was 0.36, which was significantly higher than that of older camels. In addition, the n-6/n-3 value is an important factor that affects human health and that plays a central role in epidemiology [31]. The ideal value of n-6/n-3, as recommended by the Chinese Nutrition Association, is 4–6. The results of this study showed that the value of n-6/n-3 from the ST muscle was significantly affected by age. Meat from younger camels (age group 1) had a higher n-6/n-3 value than that from older animals (age group 3), and the meat from middle-aged animals (age group 2) was in between. Concerning the muscle type, the ST muscle had higher PUFA, UFA/SFA, n-6/n-3, n-6, and n-3 values than the LT and PM muscles. However, the SFA content in the LT muscle was higher than that in the PM or ST muscles; similar results were reported by Kadim et al. [11].

The fatty acid composition of meat is a matter of great concern to consumers because it may have an impact on human health. An excessive intake of saturated fatty acids might increase the incidence of obesity, hypercholesterolemia, and cardiovascular disease [32]. However, unsaturated fatty acids and polyunsaturated fatty acids in lipids can effectively reduce serum cholesterol, especially n-6/n-3 and conjugated linoleic acid, which are beneficial to human health and have antitumor, antiatherosclerosis, and immune regulating properties [33]. From this point of view, for consumers who pay more attention to health, meat from younger camels is the best choice.

Regarding macro- and microminerals, similar to meat from other species, camel meat contains higher levels of potassium than other minerals. The mineral content in camel meat varied widely, probably because of differences in the sampling methods and muscle types [34] or because of the wide range of variability within individual animals. It was noted that age had a significant effect on the macromineral content, but not on the micromineral content (except for Fe and Cu). In this study, the meat from the 6–7 year-old camels had significantly higher P Na, Mg, and K values than the meat from younger camels (3–4 years) or older animals (9–10 years) did. This might be related to camel growth patterns. The present results showed that after reaching the age of 6–7, the mineral content in camel meat no longer increases. Concerning muscle types, the contents of Mg, K, and Zn in the ST muscle were significantly higher than those in the LT and PM muscles.

### 4.2. Effects of Age and Muscle Type on Meat Quality

The ultimate pH value is an important indicator for measuring meat quality and also has a certain impact on the tenderness of the meat [35]. The ultimate pH range of Bactrian camel meat detected in this study was between 5.49 and 6.12. In the present study, age and muscle type and age × muscle type interaction had significant effects on the ultimate muscle pH.

Meat from younger camels had a higher ultimate pH value than that from older camels (>5 years old). The higher ultimate pH value in the meat from younger camels might have resulted from differences in muscle fiber types or lower muscle glycogen in the meat [11,36]. Similarly, a study reported that younger animals tend to produce meat with higher ultimate pH values than older animals due to a lack of glycogen [36]. For the muscle types from younger camels, the ST muscle exhibited higher ultimate pH values than the PM and LT muscles did. The ultimate pH values of the LT (6.08) and ST muscles (6.12) observed in this study were higher than the values observed in Kadim’s study [25] (5.7–5.9) for the LT muscle and the value of 5.5 for the ST muscle reported by Soltanizadeh et al. [37].

For the LT muscles of dromedary camels, the pH values of 3–5 years and 6–8 years are 5.84 and 5.71, respectively. However, the current study found that pH values of camels who were 3–4 years of age and 6–7 years of age were 6.08 and 5.75, respectively. The difference between the current study and other studies may be due to a combination of several factors, including pre-slaughter handling, postmortem treatment, and metabolism of the muscles, with low muscle glycogen stores at slaughter preventing the development of a desirable pH postmortem [38]. Muscles from the >6-year-old camels were darker (lower L*) and redder (higher a*) than those from the 3–4-year-old camels, whereas no significant differences were observed in terms of the yellowness b* value between them. A darker color is likely related to the increased myoglobin content that occurs with age [25]. In addition, the type of muscle fiber and cooling method may also have an effect. Regarding muscle type, the L*, a*, and b* values of the LT and ST muscles in the present study were in agreement with those reported for dromedary camels [11]. In the present study, the LT muscles had higher L*, a*, and b* values than the ST muscles. This might be related to postmortem protein degradation increasing the light scattering properties of muscle and consequently increasing the L* value [39], which is also directly related to the ultimate pH value [39]. In addition, the oxidation of fat and the myoglobin content might be the main factors affecting the b* value of camel meat [11].

Cooking loss is one of the key characteristics of meat quality. It is closely related to the water-holding capacity and can reflect how the meat was processed [3,11,12]. A previous study reported that several factors, such as moisture, fat protein, and minerals, might affect the cooking loss and water-holding capacity [11,25]. In the current study, the cooking loss of meat from younger camels was significantly higher than that from older camels. Regarding muscle type, the PM muscles from age group 1 had a significantly higher cooking loss than the LT and ST muscles did. This might be due to the lower fat content in the PM muscle [40]. Additionally, for the LT muscles, the cooking loss value range for Bactrian camel meat was from 26.53 to 28.93% in this study, which is higher than that for dromedary camels (22.4 to 26.06%) [11].

Notably, a significant difference related to age group was observed for the shear force among the three age groups. The meat from the older camels (age group 3) was characterized by the highest shear force value (*p* < 0.05). Some studies have confirmed the finding that the shear value increases with animal age [7,11,41,42]. Shear force is an indicator of toughness, which might be associated with total protein, total collagen, and insoluble collagen content in the meat [7,14]. Sha et al. [43] reported that the values of shear force, total collagen, soluble collagen, and insoluble collagen increased with the age of the sheep, but that the collagen solubility decreased with age. The shear force values were 0.74, 0.94, and 2.56 N; the total collagen were values 3.74, 3.78, and 4.41 mg/g; the soluble collagen values were 1.84, 1.92, and 2.04 mg/g; the insoluble collagen values were 1.82, 1.94, and 2.39 mg/g; the collagen solubility values were 51.13, 48.81, and 46.12% in 3-month-, 6-month-, and 12-month-old *Longissimus thoracic* muscles, respectively. The results showed that the total collagen, soluble collagen, and insoluble collagen were significantly different between 6 months and 12 months. The shear force and collagen solubility were significantly different between the three age groups [43]. Similar results have also been reported in beef [28]. This suggests that the increase in shear force of older camels in the present study might be due to collagen.

Regarding the LT muscle from Bactrian camels, the shear force value was significantly higher for the meat from older camels (age group 3) than it was from younger animals (age group 1), while the value for the meat from middle-aged animals (age group 2) was in between. A number of studies have confirmed the findings that the shear values increase as dromedary camels become older [7,11,12]. Any difference caused by age might be related to histological changes in muscle structure and composition when the animal matures, especially in the connective tissue [11]. This suggests that the increase in the shear force of older camel meat in the present study might be related to increased connective tissue. In addition, increased collagen crosslinking is a major cause of increased meat toughness in older animals [43,44]. The pyridinoline values represent the cross-linking collagen content [45], which is significantly affected by age. Multiple studies have confirmed that older animals have a higher pyridinoline content, resulting in higher shear force values [43,45,46]. Beef [46] from 1.5-year-old animals contains 15.00 μmol/g pyridinoline and 9.57 kg shear force values, which are significantly lower than those from 4.5-year-old animals (22.08 μmol/g, 14.95 kg, respectively). Additionally, for mutton [43], 3-month-old animals showed a pyridinoline content of 0.74 μmol/L and shear force values of 1.61 N, both of which were significantly lower than those from 12-month-old animals (2.56 μmol /L, 1.99 N, respectively). However, studies on the effects of age on collagen and collagen cross-linking in camel meat are particularly scarce.

Muscle type had a significant effect on shear force for the three age groups; the ST muscles had significantly higher values than those observed in the LT and PM muscle samples, and no significant differences were noted between the latter two muscles in the present study. These differences may have been due to variations in soluble collagen content, fiber types, and proteolytic activity [11,43,46,47]. In 12-month-old mutton [43], the total collagen (9.62, 4.41 mg/g), soluble collagen (3.64, 2.04 mg/g), insoluble collagen (5.98, 2.39 mg/g), collagen solubility (38.14, 41.12%), and pyridinoline content (2.96, 2.56 μmol/L) between the *biceps femoris* and *Longissimus thoracic* were all significantly different (*p* < 0.05). Additionally, the results showed that the shear force of the BF muscle sample (2.45N) was significantly higher than that of the ST muscle sample (1.99N). Similar results were also reported in beef [46], in which the collagen and pyridinoline content in the *Longissimus thoracic* and *Psoas major* muscles were significantly lower than those in the *biceps femoris* and *semimembranosus,* and the shear force values were also lower. From these experimental data, it can be seen that the differences in the shear force between muscle types might be related to the cross-linking collagen (pyridinoline content) and collagen solubility. The proportion of Type I oxidative, Type IIA high oxidative, and Type IIB fiber types were 33.1%, 25.2%, and 41.7% in dromedary camel LT muscles [25]. In addition, it was reported that the proportion of Type I fibers was significantly higher in the ST muscles than it was in the LT muscles [11]. However, no significant differences were found for the shear force values between the ST and PM muscles in the present study.

## 5. Conclusions

This study indicated that young camel meat (less than 4 years of age) is characterized by low fat, high moisture, and high protein contents and that it is a good source of amino acids, unsaturated fatty acids, and minerals. Moreover, it exhibits desirable meat quality characteristics, with superior tenderness and juiciness. The *Longissimus thoracic* muscle has a higher fat content and better tenderness than the *Semitendinosus* and *Psoas major* muscle, and it has been proven to be one of the most suitable muscles for marketing and processing. Traditionally, consumers in China have viewed camel meat as being unacceptably tough, mainly because the slaughtered animals are generally 8 years of age and above, resulting in poor quality characteristics. In the present study, the results showed that the age of the camel had a vital effect on the composition and quality of the meat and should be taken into consideration when slaughtering camels for meat consumption. Meanwhile, the chemical composition and quality characteristics analyzed in Bactrian camel meat introduce new knowledge about the marketing and processing of camel meat and provide more information about the meat quality and the optimal slaughter age.

## Figures and Tables

**Table 1:** Mean moisture, protein, fat, and ash components for the *Longissimus thoracic* (LT), *Semitendinosus* (ST), and *Psoas major* (PM) muscle samples from Alxa Bactrian camels (*n* = 5, mean ± standard error) slaughtered at three different ages.

Chemical Composition%	Age(Year)	Muscle		
		PM	LT	ST
Moisture	3–4	73.92 ± 0.77 ^ax^	74.52 ± 1.31 ^ax^	75.75 ± 1.33 ^ax^
	6–7	73.15 ± 0.85 ^ax^	71.23 ± 3.28 ^aby^	73.38 ± 1.40 ^bx^
	9–10	72.45 ± 1.02 ^ax^	70.89 ± 3.53 ^by^	73.10 ± 1.96 ^bx^
	Effect	*P*_age_ < 0.05	*P*_muscle_ < 0.05	*P*_age×muscle_ = 0.16
Protein	3–4	19.60 ± 1.44 ^bx^	19.78 ± 2.14 ^bx^	20.56 ± 0.91 ^bx^
	6–7	21.28 ± 0.26 ^abx^	21.15 ± 0.79 ^abx^	21.90 ± 0.91 ^ax^
	9–10	21.45 ± 1.18 ^ax^	22.42 ± 1.12 ^ax^	22.33 ± 0.45 ^ax^
	Effect	*P*_age_ < 0.05	*P*_muscle_ = 0.26	*P_a_*_ge×muscle_ = 0.73
Crude fat	3–4	1.22 ± 0.23 ^by^	3.66 ± 1.22 ^bx^	0.40 ± 0.12 ^bz^
	6–7	1.93 ± 0.89 ^by^	4.20 ± 0.55 ^bx^	1.40 ± 1.03 ^abz^
	9–10	4.38 ± 2.78 ^ay^	7.78 ± 2.49 ^ax^	2.23 ± 1.55 ^az^
	Effect	*P*_age_ < 0.05	*P*_muscle_ < 0.05	*P*_age×muscle_ = 0.33
Ash	3–4	1.11 ± 0.09 ^ax^	1.13 ± 0.02 ^ax^	1.15 ± 0.03 ^ax^
	6–7	1.19 ± 0.02 ^ax^	1.19 ± 0.25 ^ax^	1.14 ± 0.12 ^ax^
	9–10	1.14 ± 0.03 ^ax^	1.39 ± 0.29 ^ax^	1.23 ± 0.28 ^ax^
	Effect	*P*_age_ = 0.16	*P*_muscle_ = 0.32	*P*_age×muscle_ = 0.45
Moisture: protein ratio	3–4	3.62 ± 0.26 ^ax^	3.80 ± 0.44 ^ax^	3.69 ± 0.14 ^ax^
	6–7	3.44 ± 0.05 ^ax^	3.27 ± 0.27 ^bx^	3.35 ± 0.15 ^abx^
	9–10	3.46 ± 0.19 ^ax^	3.10 ± 0.26 ^bx^	3.31 ± 0.25 ^bx^
	Effect	*P*_age_ < 0.05	*P*_muscle_ = 0.41	*P*_age×muscle_ = 0.22

^a,b^ The variables with the same letters in the same column do not differ (*p* ≥ 0.05). ^x–z^ The variables with the same letters in the same row do not differ (*p* ≥ 0.05). *P*_age_, *P*_muscle_, and *P*_age×muscle_ are the effects of age, muscle type, and the age × muscle type interaction on the chemical composition of the meat, respectively.

**Table 2:** Comparison of the amino acid content in *Longissimus thoracic* (LT), *Semitendinosus* (ST), and *Psoas major* (PM) muscle samples from Alxa Bactrian camels (*n* = 5, mean ± standard error) slaughtered at three different ages.

Amino Acids (g/100 g)	Age (Year)	Muscle		
		PM	LT	ST
Asp	3–4	1.91 ± 0.07 ^ax^	1.81 ± 0.15 ^bx^	1.88 ± 0.13 ^ax^
	6–7	1.97 ± 0.10 ^ax^	1.89 ± 0.14 ^abx^	1.94 ± 0.15 ^ax^
	9–10	1.95 ± 0.14 ^ax^	2.05 ± 0.11 ^ax^	2.05 ± 0.15 ^ax^
	Effect	*P*_age_ < 0.05	*P*_muscle_ = 0.67	*P*_age×muscle_ = 0.50
Thr	3–4	0.94 ± 0.03 ^ax^	0.89 ± 0.08 ^ax^	0.92 ± 0.06 ^ax^
	6–7	0.97 ± 0.04 ^ax^	0.91 ± 0.07 ^ax^	0.94 ± 0.07 ^ax^
	9–10	0.95 ± 0.06 ^ax^	0.99 ± 0.06 ^ax^	0.99 ± 0.08 ^ax^
	Effect	*P*_age_ < 0.05	*P*_muscle_ = 0.50	*P*_age×muscle_ = 0.46
Ser	3–4	0.81 ± 0.03 ^ax^	0.77 ± 0.07 ^ax^	0.79 ± 0.05 ^ax^
	6–7	0.82 ± 0.05 ^ax^	0.78 ± 0.07 ^ax^	0.82 ± 0.06 ^ax^
	9–10	0.82 ± 0.04 ^ax^	0.85 ± 0.05 ^ax^	0.86 ± 0.07 ^ax^
	Effect	*P*_age_ = 0.05	*P*_muscle_ = 0.55	*P*_age×muscle_ = 0.48
Glu	3–4	3.09 ± 0.13 ^ax^	2.95 ± 0.26 ^ax^	3.08 ± 0.18 ^ax^
	6–7	3.17 ± 0.16 ^ax^	2.99 ± 0.26 ^ax^	3.11 ± 0.25 ^ax^
	9–10	3.15 ± 0.18 ^ax^	3.29 ± 0.17 ^ax^	3.34 ± 0.22 ^ax^
	Effect	*P*_age_ < 0.05	*P*_muscle_ = 0.40	*P*_age×muscle_ = 0.46
Gly	3–4	0.81 ± 0.04 ^ax^	0.77 ± 0.06 ^ax^	0.83 ± 0.07 ^bx^
	6–7	0.86 ± 0.04 ^ax^	0.83 ± 0.10 ^ax^	0.86 ± 0.05 ^bx^
	9–10	0.84 ± 0.05 ^ax^	0.89 ± 0.06 ^ax^	0.96 ± 0.05 ^ax^
	Effect	*P*_age_ < 0.05	*P*_muscle_ = 0.19	*P*_age×muscle_ = 0.53
Ala	3–4	1.13 ± 0.05 ^ax^	1.07 ± 0.09 ^bx^	1.11 ± 0.07 ^bx^
	6–7	1.16 ± 0.05 ^ax^	1.12 ± 0.11 ^abx^	1.15 ± 0.08 ^abx^
	9–10	1.15 ± 0.07 ^ax^	1.21 ± 0.07 ^ax^	1.26 ± 0.04 ^ax^
	Effect	*P*_age_ < 0.05	*P*_muscle_ = 0.56	*P*_age×muscle_ = 0.52
Val	3–4	0.95 ± 0.05 ^ax^	0.90 ± 0.08 ^bx^	0.94 ± 0.08 ^ax^
	6–7	1.00 ± 0.04 ^ax^	0.96 ± 0.07 ^abx^	0.99 ± 0.06 ^ax^
	9–10	0.99 ± 0.05 ^ax^	1.05 ± 0.05 ^ax^	1.04 ± 0.09 ^ax^
	Effect	*P*_age_ < 0.05	*P*_muscle_ = 0.78	*P*_age×muscle_ = 0.37
Met	3–4	0.55 ± 0.03 ^ax^	0.52 ± 0.05 ^bx^	0.54 ± 0.04 ^ax^
	6–7	0.56 ± 0.04 ^ax^	0.53 ± 0.05 ^abx^	0.54 ± 0.04 ^ax^
	9–10	0.54 ± 0.07 ^ax^	0.59 ± 0.03 ^ax^	0.56 ± 0.07 ^ax^
	Effect	*P*_age_ = 0.38	*P*_muscle_ = 0.98	*P*_age×muscle_ = 0.42
Ileu	3–4	0.88 ± 0.04 ^ax^	0.84 ± 0.08 ^bx^	0.88 ± 0.07 ^ax^
	6–7	0.93 ± 0.04 ^ax^	0.87 ± 0.07 ^bx^	0.91 ± 0.08 ^ax^
	9–10	0.91 ± 0.39 ^ax^	0.97 ± 0.04 ^ax^	0.96 ± 0.09 ^ax^
	Effect	*P*_age_ < 0.05	*P*_muscle_ = 0.55	*P*_age×muscle_ = 0.36
Leu	3–4	1.67 ± 0.07 ^ax^	1.58 ± 0.13 ^bx^	1.64 ± 0.11 ^ax^
	6–7	1.75 ± 0.09 ^ax^	1.64 ± 0.14 ^abx^	1.71 ± 0.13 ^ax^
	9–10	1.72 ± 0.11 ^ax^	1.79 ± 0.09 ^ax^	1.81 ± 0.13 ^ax^
	Effect	*P*_age_ < 0.05	*P*_muscle_ = 0.43	*P*_age×muscle_ = 0.48
Tyr	3–4	0.73 ± 0.03 ^ax^	0.68 ± 0.06 ^bx^	0.71 ± 0.05 ^ax^
	6–7	0.75 ± 0.04 ^ax^	0.70 ± 0.06 ^abx^	0.74 ± 0.07 ^ax^
	9–10	0.73 ± 0.06 ^ax^	0.78 ± 0.03 ^ax^	0.78 ± 0.07 ^ax^
	Effect	*P*_age_ < 0.05	*P*_muscle_ = 0.55	*P*_age×muscle_ = 0.36
Phe	3–4	0.8 ± 0.04 ^ax^	0.75 ± 0.07 ^bx^	0.77 ± 0.05 ^bx^
	6–7	0.81 ± 0.04 ^ax^	0.79 ± 0.06 ^abx^	0.83 ± 0.07 ^abx^
	9–10	0.82 ± 0.05 ^ax^	0.86 ± 0.03 ^ax^	0.87 ± 0.05 ^ax^
	Effect	*P*_age_ < 0.05	*P*_muscle_ = 0.45	*P*_age×muscle_ = 0.46
His	3–4	1.06 ± 0.09 ^ax^	1.03 ± 0.09 ^bx^	1.03 ± 0.06 ^ax^
	6–7	1.13 ± 0.03 ^ax^	1.10 ± 0.06 ^abx^	1.12 ± 0.05 ^ax^
	9–10	1.06 ± 0.09 ^ax^	1.13 ± 0.03 ^ax^	1.06 ± 0.16 ^ax^
	Effect	*P*_age_ < 0.05	*P*_muscle_ = 0.88	*P*_age×muscle_ = 0.53
Lys	3–4	1.81 ± 0.08 ^ax^	1.72 ± 0.14 ^bx^	1.79 ± 0.12 ^ax^
	6–7	1.85 ± 0.08 ^ax^	1.79 ± 0.16 ^abx^	1.84 ± 0.14 ^ax^
	9–10	1.84 ± 0.11 ^ax^	1.95 ± 0.11 ^ax^	1.93 ± 0.14 ^ax^
	Effect	*P*_age_ < 0.05	*P*_muscle_ = 0.69	*P*_age×muscle_ = 0.51
Arg	3–4	1.24 ± 0.05 ^ax^	1.17 ± 0.12 ^bx^	1.24 ± 0.09 ^ax^
	6–7	1.27 ± 0.04 ^ax^	1.22 ± 0.11 ^abx^	1.25 ± 0.10 ^ax^
	9–10	1.28 ± 0.08 ^ax^	1.36 ± 0.10 ^ax^	1.35 ± 0.10 ^ax^
	Effect	*P*_age_ < 0.05	*P*_muscle_ = 0.66	*P*_age×muscle_ = 0.47
Pro	3–4	0.67 ± 0.06 ^ax^	0.64 ± 0.06 ^bx^	0.66 ± 0.03 ^bx^
	6–7	0.71 ± 0.08 ^ax^	0.65 ± 0.05 ^bx^	0.69 ± 0.03 ^abx^
	9–10	0.71 ± 0.04 ^ax^	0.75 ± 0.06 ^ax^	0.74 ± 0.06 ^ax^
	Effect	*P*_age_ < 0.05	*P*_muscle_ = 0.69	*P*_age×muscle_ = 0.41
TAA	3–4	19.03 ± 0.81 ^ax^	18.09 ± 1.57 ^bx^	18.35 ± 0.53 ^bx^
	6–7	19.69 ± 0.88 ^ax^	18.79 ± 1.51 ^abx^	19.46 ± 1.38 ^bx^
	9–10	19.48 ± 1.17 ^ax^	20.50 ± 1.07 ^ax^	21.19 ± 0.44 ^ax^
	Effect	*P*_age_ < 0.05	*P*_muscle_ = 0.59	*P*_age×muscle_ = 0.44
EAA	3–4	7.59 ± 0.33 ^ax^	7.20 ± 0.63 ^bx^	7.27 ± 0.18 ^bx^
	6–7	7.86 ± 0.35 ^ax^	7.49 ± 0.62 ^abx^	7.77 ± 0.57 ^bx^
	9–10	7.78 ± 0.47 ^ax^	8.19 ± 0.39 ^ax^	8.45 ± 0.18 ^ax^
	Effect	*P*_age_ < 0.05	*P*_muscle_ = 0.62	*P*_age×muscle_ = 0.44
NEAA	3–4	11.44 ± 0.48 ^ax^	10.90 ± 0.95 ^ax^	11.08 ± 0.36 ^bx^
	6–7	11.83 ± 0.53 ^ax^	11.30 ± 0.91 ^ax^	11.69 ± 0.81 ^bx^
	9–10	11.69 ± 0.70 ^ax^	12.31 ± 0.68 ^ax^	12.75 ± 0.29 ^ax^
	Effect	*P*_age_ < 0.05	*P*_muscle_ = 0.58	*P*_age×muscle_ = 0.44
EAA/TAA	3–4	39.88 ± 0.06 ^ax^	39.77 ± 0.15 ^ax^	39.61 ± 0.28 ^ax^
	6–7	39.92 ± 0.25 ^ax^	39.87 ± 0.45 ^ax^	39.92 ± 0.24 ^ax^
	9–10	39.97 ± 0.20 ^ax^	39.95 ± 0.28 ^ax^	39.86 ± 0.36 ^ax^
	Effect	*P*_age_ = 0.45	*P*_muscle_ = 0.65	*P*_age×muscle_ = 0.96
EAA/NEAA	3–4	66.33 ± 0.17 ^ax^	66.03 ± 0.43 ^ax^	65.59 ± 0.76 ^ax^
	6–7	66.47 ± 0.69 ^ax^	66.32 ± 1.25 ^ax^	66.46 ± 0.66 ^ax^
	9–10	66.58 ± 0.56 ^ax^	66.53 ± 0.79 ^ax^	66.28 ± 1.00 ^ax^
	Effect	*P*_age_ = 0.44	*P*_muscle_ = 0.67	*P*_age×muscle_ = 0.96

^a,b^ The variables with the same letters in the same column do not differ (*p* ≥ 0.05). ^x–z^ The variables with the same letters in the same row do not differ (*p* ≥ 0.05). *P*_age_, *P*_muscle_, and *P*_age×muscle_ are the effects of age, muscle type, and the age × muscle type interaction on the amino acids of meat, respectively. His, histidine; Ileu, isoleucine; Leu, leucine; Lys, lysine; Met, methionine; Phe, phenylalanine; Thr, threonine; Try, tryptophane; Val, valine; Glu, glutamic acid; Pro, proline; Tyr, tyrosine; Ala, alanine; Arg, arginine; Ser, serine; Gly, glycine; Asp, aspartic acid. TAA, total amino acids; EAA, essential amino acids; NEAA, non-essential amino acids.

**Table 3:** Macro and micro-element levels (mg/100 g) in the *Longissimus thoracic* (LT), *Semitendinosus* (ST), and *Psoas major* (PM) muscle samples from Alxa Bactrian camels (*n* = 5, mean ± standard error) slaughtered at three different ages.

Component	Age Group (Year)	Muscle		
		PM	LT	ST
Calcium (Ca)	3–4	4.33 ± 0.55 ^ax^	3.69 ± 0.17 ^bx^	4.59 ± 0.19 ^ax^
	6–7	4.40 ± 0.37 ^ax^	4.62 ± 0.61 ^ax^	4.37 ± 0.42 ^ax^
	9–10	4.89 ± 0.38 ^ax^	4.53 ± 0.69 ^abx^	4.33 ± 0.23 ^ax^
	Effect	P_age_ = 0.49	P_muscle_ = 0.84	P_age×muscle_ = 0.75
Phosphorus (P)	3–4	219.00 ± 5.87 ^ax^	204.50 ± 6.856 ^bx^	204.40 ± 13.334 ^bx^
	6–7	232.25 ± 9.81 ^ax^	226.00 ± 10.668 ^ax^	230.60 ± 14.673 ^ax^
	9–10	230.80 ± 9.78 ^ax^	202.00 ± 7.563 ^abx^	227.00 ± 17.720 ^abx^
	Effect	*P*_age_ < 0.05	*P*_muscle_*=* 0.17	*P*_age×muscle_ = 0.87
Sodium (Na)	3–4	54.45 ± 4.69 ^ax^	52.68 ± 5.41 ^ax^	55.56 ± 6.11 ^ax^
	6–7	52.38 ± 8.99 ^ax^	50.20 ± 4.95 ^ax^	46.48 ± 6.48 ^ax^
	9–10	57.72 ± 6.68 ^ax^	45.74 ± 5.35 ^ay^	48.05 ± 7.54 ^ay^
	Effect	*P*_age_ = 0.27	*P*_muscle_ = 0.25	*P*_age×muscle_ = 0.34
Magnesium (Mg)	3–4	23.82 ± 1.89 ^ax^	22.33 ± 0.98 ^ax^	22.98 ± 1.20 ^ax^
	6–7	25.08 ± 1.35 ^ax^	24.12 ± 1.72 ^ay^	25.26 ± 2.02 ^ax^
	9–10	25.18 ± 1.02 ^ax^	23.12 ± 1.01 ^ax^	24.73 ± 1.53 ^ax^
	Effect	*P*_age_ = 0.06	*P*_muscle_ = 0.14	*P*_age×muscle_ = 0.81
Potassium (K)	3–4	381.20 ± 10.14 ^bx^	358.25 ± 11.50 ^bx^	368.40 ± 12.43 ^ax^
	6–7	412.75 ± 11.01 ^axy^	402.60 ± 12.47 ^ay^	422.80 ± 15.29 ^ax^
	9–10	401.20 ± 7.33 ^abx^	377.40 ± 11.22 ^abx^	425.25 ± 13.82 ^ax^
	Effect	*P*_age_ < 0.05	*P*_muscle_ = 0.47	*P*_age×muscle_ = 0.93
Zinc (Zn)	3–4	2.521 ± 0.588 ^ax^	3.032 ± 0.252 ^ax^	2.731 ± 0.588 ^bx^
	6–7	2.653 ± 0.697 ^ay^	3.113 ± 0.200 ^axy^	3.443 ± 0.391 ^ax^
	9–10	2.644 ± 0.470 ^ax^	2.942 ± 0.141 ^ax^	2.691 ± 0.155 ^bx^
	Effect	*P*_age_ = 0.54	*P*_muscle_*=* 0.08	*P*_age×muscle_ = 0.67
Iron (Fe)	3–4	1.442 ± 0.264 ^bx^	1.202 ± 0.102 ^bx^	1.282 ± 0.427 ^bx^
	6–7	1.851 ± 0.596 ^abx^	1.844 ± 0.473 ^ax^	2.351 ± 0.649 ^ax^
	9–10	2.201 ± 0.384 ^ax^	1.823 ± 0.341 ^ax^	1.674 ± 0.272 ^abx^
	Effect	*P*_age_ < 0.05	*P*_muscle_*=* 0.51	*P*_age×muscle_ = 0.36
Lead (Pb)	3–4	0.003 ± 0.001 ^ax^	0.003 ± 0.000 ^ax^	0.001 ± 0.000 ^ax^
	6–7	0.001 ± 0.000 ^ax^	0.002 ± 0.000 ^ax^	0.002 ± 0.000 ^ax^
	9–10	0.002 ± 0.000 ^ax^	0.002 ± 0.000 ^ax^	0.003 ± 0.001 ^ax^
	Effect	*P*_age_ < 0.05	*P*_muscle_*=* 0.39	*P*_age×muscle_ = 0.78
Selenium (Se)	3–4	0.008 ± 0.001 ^ax^	0.009 ± 0.001 ^ax^	0.008 ± 0.001 ^ax^
	6–7	0.009 ± 0.000 ^ax^	0.010 ± 0.001 ^ax^	0.010 ± 0.000 ^ax^
	9–10	0.009 ± 0.001 ^ax^	0.010 ± 0.011 ^ax^	0.009 ± 0.001 ^ax^
	Effect	*P*_age_ < 0.05	*P*_muscle_ = 0.28	*P*_age×muscle_ = 0.86
Copper (Cu)	3–4	0.187 ± 0.024 ^ax^	0.175 ± 0.013 ^ax^	0.145 ± 0.071 ^ax^
	6–7	0.086 ± 0.003 ^ax^	0.081 ± 0.009 ^abx^	0.123 ± 0.067 ^ax^
	9–10	0.105 ± 0.059 ^ax^	0.056 ± 0.009 ^bx^	0.071 ± 0.002 ^ax^
	Effect	*P*_age_ < 0.05	*P*_muscle_ = 0.48	*P*_age×muscle_ = 0.79

^a,b^ The variables with the same letters in the same column do not differ (*p* ≥ 0.05). ^x–z^ The variables with the same letters in the same row do not differ (*p* ≥ 0.05). *P*_age_, *P*_muscle_, and *P*_age×muscle_ are effects of age, muscle type, and the age × muscle type interaction on the macro and micro-elements of meat, respectively.

**Table 4:** Fatty acid composition (%) of the *Psoas major* (PM), *Longissimus thoracic* (LT), and *semitendinosus* (ST) muscle samples from Alxa Bactrian camels (*n* = 5, mean ± standard error) slaughtered at three different ages.

Fatty Acid	Age (Year)	Muscle		
		PM	LT	ST
Saturated fatty acid				
C4:0	3–4	0.06 ± 0.01 ^ay^	0.03 ± 0.01 ^ay^	0.18 ± 0.12 ^ax^
	6–7	0.03 ± 0.01 ^ax^	0.01 ± 0.00 ^ax^	0.05 ± 0.04 ^bx^
	9–10	0.06 ± 0.05 ^ax^	0.02 ± 0.01 ^ax^	0.07 ± 0.05 ^abx^
	Effect	*P*_age_ < 0.05	*P*_muscle_ < 0.05	*P*_age×muscle_ < 0.05
C6:0	3–4	0.01 ± 0.00	ND	0.02 ± 0.01
	6–7	0.01 ± 0.00	ND	0.01 ± 0.00
	9–10	0.02 ± 0.03	ND	0.24 ± 0.02
	Effect	*P*_age_ = 0.28	*P*_muscle_ = 0.14	*P*_age×muscle_ = 0.67
C8:0	3–4	0.02 ± 0.01 ^ax^	0.05 ± 0.03 ^ax^	0.02 ± 0.01 ^ax^
	6–7	0.03 ± 0.01 ^ax^	0.03 ± 0.00 ^ax^	0.02 ± 0.01 ^ax^
	9–10	0.03 ± 0.02 ^ax^	0.03 ± 0.01 ^ax^	0.02 ± 0.01 ^ax^
	Effect	*P*_age_ = 0.39	*P*_muscle_ < 0.05	*P*_age×muscle_ = 0.47
C10:0	3–4	0.15 ± 0.04 ^ax^	0.18 ± 0.09 ^ax^	0.26 ± 0.12 ^ax^
	6–7	0.12 ± 0.04 ^ax^	0.14 ± 0.04 ^ax^	0.12 ± 0.03 ^bx^
	9–10	0.15 ± 0.09 ^ax^	0.12 ± 0.05 ^ax^	0.14 ± 0.02 ^bx^
	Effect	*P*_age_ < 0.05	*P*_muscle_ = 0.18	*P*_age×muscle_ = 0.11
C11:0	3–4	ND	ND	ND
	6–7	ND	ND	ND
	9–10	ND	ND	ND
C12:0	3–4	0.28 ± 0.03 ^ax^	0.42 ± 0.20 ^ax^	0.25 ± 0.03 ^ax^
	6–7	0.38 ± 0.15 ^ax^	0.37 ± 0.06 ^ax^	0.27 ± 0.04 ^ax^
	9–10	0.43 ± 0.24 ^ax^	0.42 ± 0.14 ^ax^	0.36 ± 0.15 ^ax^
	Effect	*P*_age_ = 0.21	*P*_muscle_ = 0.12	*P*_age×muscle_ = 0.72
C13:0	3–4	0.02 ± 0.01 ^axy^	0.03 ± 0.02 ^ax^	0.01 ± 0.00 ^ay^
	6–7	0.03 ± 0.01 ^ax^	0.03 ± 0.01 ^ax^	0.01 ± 0.00 ^ax^
	9–10	0.03 ± 0.01 ^ax^	0.02 ± 0.01 ^ax^	0.02 ± 0.01 ^ax^
	Effect	*P*_age_ = 0.65	*P*_muscle_ < 0.05	*P*_age×muscle_ = 0.43
C14:0	3–4	5.50 ± 0.56 ^ay^	7.54 ± 1.59 ^ax^	4.84 ± 1.29 ^by^
	6–7	7.34 ± 1.93 ^ax^	7.75 ± 0.57 ^ax^	6.56 ± 1.09 ^abx^
	9–10	7.77 ± 2.12 ^ax^	9.08 ± 2.17 ^ax^	7.87 ± 2.00 ^ax^
	Effect	*P*_age_ < 0.05	*P*_muscle_ < 0.05	*P*_age×muscle_ = 0.73
C15:0	3–4	0.61 ± 0.12 ^axy^	0.71 ± 0.21 ^ax^	0.42 ± 0.06 ^ay^
	6–7	0.75 ± 0.09 ^ax^	0.68 ± 0.19 ^axy^	0.50 ± 0.05 ^ay^
	9–10	0.68 ± 0.22 ^ax^	0.65 ± 0.19 ^ax^	0.54 ± 0.26 ^ax^
	Effect	*P*_age_ = 0.45	*P*_muscle_ < 0.05	*P*_age×muscle_ = 0.62
C16:0	3–4	25.10 ± 1.15 ^bx^	26.40 ± 0.95 ^cx^	22.22 ± 1.96 ^by^
	6–7	30.99 ± 2.35 ^ax^	30.33 ± 1.07 ^bx^	28.16 ± 4.21 ^ax^
	9–10	32.46 ± 2.07 ^ax^	34.03 ± 1.4 ^ax^	29.66 ± 4.27 ^ax^
	Effect	*P*_age_ < 0.05	*P*_muscle_ < 0.05	*P*_age×muscle_ = 0.82
C17:0	3–4	0.80 ± 0.12 ^ax^	0.88 ± 0.18 ^ax^	0.57 ± 0.05 ^ay^
	6–7	0.84 ± 0.11 ^ax^	0.73 ± 0.15 ^axy^	0.57 ± 0.11 ^ay^
	9–10	0.76 ± 0.18 ^ax^	0.69 ± 0.16 ^ax^	0.53 ± 0.17 ^ax^
	Effect	*P*_age_ = 0.26	*P*_muscle_ < 0.05	*P*_age×muscle_ = 0.61
C18:0	3–4	20.59 ± 2.11 ^ax^	21.12 ± 3.79 ^ax^	14.25 ± 1.84 ^ay^
	6–7	17.29 ± 5.46 ^abx^	16.14 ± 4.80 ^abx^	12.52 ± 1.67 ^abx^
	9–10	13.67 ± 3.14 ^bx^	13.43 ± 2.93 ^bx^	9.89 ± 1.98 ^bx^
	Effect	*P*_age_ < 0.05	*P*_muscle_ < 0.05	*P*_age×muscle_ = 0.76
C20:0	3–4	0.28 ± 0.03 ^ax^	0.26 ± 0.06 ^ax^	0.18 ± 0.02 ^ay^
	6–7	0.24 ± 0.08 ^ax^	0.23 ± 0.06 ^ax^	0.18 ± 0.02 ^ax^
	9–10	0.19 ± 0.06 ^ax^	0.19 ± 0.05 ^ax^	0.13 ± 0.02 ^bx^
	Effect	*P*_age_ < 0.05	*P*_muscle_ < 0.05	*P*_age×muscle_ = 0.82
C21:0	3–4	0.55 ± 0.06 ^ax^	0.66 ± 0.07 ^ax^	0.59 ± 0.16 ^ax^
	6–7	0.77 ± 0.25 ^ax^	0.92 ± 0.29 ^ax^	0.84 ± 0.27 ^ax^
	9–10	0.68 ± 0.22 ^ax^	0.67 ± 0.17 ^ax^	0.62 ± 0.13 ^ax^
	Effect	*P*_age_ < 0.05	*P*_muscle_ = 0.53	*P*_age×muscle_ = 0.92
C22:0	3–4	0.11 ± 0.02 ^axy^	0.06 ± 0.02 ^ay^	0.15 ± 0.05 ^ax^
	6–7	0.06 ± 0.02 ^bx^	0.05 ± 0.006 ^ax^	0.08 ± 0.04 ^bx^
	9–10	0.05 ± 0.02 ^bx^	0.36 ± 0.02 ^ax^	0.05 ± 0.03 ^bx^
	Effect	*P*_age_ < 0.05	*P*_muscle_ < 0.05	*P*_age×muscle_ < 0.05
C23:0	3–4	1.65 ± 0.40 ^ay^	0.52 ± 0.14 ^az^	3.06 ± 1.11 ^ax^
	6–7	0.68 ± 0.03 ^bxy^	0.49 ± 0.33 ^ay^	1.56 ± 0.86 ^abx^
	9–10	0.85 ± 0.39 ^bxy^	0.34 ± 0.08 ^ay^	1.41 ± 0.93 ^bx^
	Effect	*P*_age_ < 0.05	*P*_muscle_ < 0.05	*P*_age×muscle_ = 0.06
C24:0	3–4	0.07 ± 0.01 ^ay^	0.03 ± 0.01 ^ay^	0.12 ± 0.03 ^ax^
	6–7	0.03 ± 0.02 ^bx^	0.03 ± 0.01 ^ax^	0.06 ± 0.04 ^abx^
	9–10	0.03 ± 0.02 ^bx^	0.02 ± 0.01 ^ax^	0.03 ± 0.02 ^bx^
	Effect	*P*_age_ < 0.05	*P*_muscle_ < 0.05	*P*_age×muscle_ < 0.05
Mono-unsaturated fatty acids				
C14:1	3–4	0.09 ± 0.03 ^ax^	0.14 ± 0.06 ^ax^	0.14 ± 0.07 ^bx^
	6–7	0.12 ± 0.05 ^ax^	0.15 ± 0.03 ^ax^	0.22 ± 0.05 ^abx^
	9–10	0.15 ± 0.16 ^ax^	0.23 ± 0.11 ^ax^	0.31 ± 0.09 ^ax^
	Effect	*P*_age_ < 0.05	*P*_muscle_ = 0.09	*P*_age×muscle_ = 0.85
C15:1	3–4	ND	ND	ND
	6–7	ND	ND	ND
	9–10	ND	ND	ND
C16:1	3–4	2.47 ± 0.39 ^bx^	2.81 ± 0.52 ^ax^	2.98 ± 0.79 ^bx^
	6–7	3.54 ± 0.77 ^ax^	4.20 ± 1.79 ^ax^	4.12 ± 0.33 ^ax^
	9–10	3.95 ± 0.64 ^ax^	4.11 ± 0.81 ^ax^	4.83 ± 0.91 ^ax^
	Effect	*P*_age_ < 0.05	*P*_muscle_ = 0.15	*P*_age×muscle_ = 0.88
C17:1	3–4	0.41 ± 0.02 ^bx^	0.45 ± 0.07 ^ax^	0.45 ± 0.07 ^ax^
	6–7	0.50 ± 0.05 ^abx^	0.49 ± 0.05 ^ax^	0.52 ± 0.05 ^ax^
	9–10	0.51 ± 0.07 ^ax^	0.48 ± 0.06 ^ax^	0.53 ± 0.11 ^ax^
	Effect	*P*_age_ < 0.05	*P*_muscle_ = 0.56	*P*_age×muscle_ = 0.87
C18:1n9t	3–4	0.31 ± 0.02 ^ax^	0.28 ± 0.11 ^ax^	0.37 ± 0.13 ^ax^
	6–7	0.35 ± 0.06 ^ax^	0.23 ± 0.13 ^ax^	0.29 ± 0.04 ^ax^
	9–10	0.29 ± 0.08 ^axy^	0.19 ± 0.07 ^ay^	0.31 ± 0.04 ^ax^
	Effect	*P*_age_ = 0.24	*P*_muscle_ < 0.05	*P*_age×muscle_ = 0.67
C18:1n9c	3–4	30.67 ± 1.25 ^ax^	31.12 ± 1.59 ^ax^	31.58 ± 3.25 ^ax^
	6–7	29.97 ± 0.91 ^ay^	32.25 ± 2.17 ^axy^	34.30 ± 2.26 ^ax^
	9–10	29.99 ± 1.91 ^ax^	31.05 ± 2.83 ^ax^	34.10 ± 4.63 ^ax^
	Effect	*P*_age_ = 0.56	*P*_muscle_ < 0.05	*P*_age×muscle_ = 0.56
C20:1	3–4	0.22 ± 0.03 ^ax^	0.21 ± 0.05 ^ax^	0.26 ± 0.04 ^ax^
	6–7	0.21 ± 0.04 ^ay^	0.26 ± 0.06 ^axy^	0.31 ± 0.05 ^ax^
	9–10	0.25 ± 0.05 ^ax^	0.26 ± 0.04 ^ax^	0.32 ± 0.09 ^ax^
	Effect	*P*_age_ = 0.08	*P*_muscle_ < 0.05	*P*_age×muscle_ = 0.75
C22:1	3–4	0.07 ± 0.17 ^ay^	0.04 ± 0.01 ^ay^	0.27 ± 0.12 ^ax^
	6–7	0.05 ± 0.17 ^bx^	0.04 ± 0.01 ^ax^	0.06 ± 0.04 ^bx^
	9–10	0.38 ± 0.16 ^bx^	0.02 ± 0.01 ^ax^	0.06 ± 0.05 ^bx^
	Effect	*P*_age_ < 0.05	*P*_muscle_ < 0.05	*P*_age×muscle_ < 0.05
C24:1	3–4	0.03 ± 0.01 ^ax^	0.02 ± 0.01 ^abx^	0.03 ± 0.01 ^ax^
	6–7	0.04 ± 0.02 ^ax^	0.04 ± 0.01 ^ax^	0.05 ± 0.02 ^ax^
	9–10	0.02 ± 0.01 ^ax^	0.02 ± 0.01 ^bx^	0.02 ± 0.01 ^ax^
	Effect	*P*_age_ < 0.05	*P*_muscle_ = 0.70	*P*_age×muscle_ = 0.96
Poly-unsaturated fatty acids				
C18:2n6t	3–4	0.06 ± 0.01 ^ax^	0.03 ± 0.02 ^ax^	ND
	6–7	0.05 ± 0.02 ^ax^	0.03 ± 0.01 ^axy^	0.01 ± 0.00 ^ay^
	9–10	0.06 ± 0.05 ^ax^	0.02 ± 0.01 ^ax^	0.02 ± 0.01 ^ax^
	Effect	*P*_age_ = 0.93	*P*_muscle_ = 0.05	*P*_age×muscle_ = 0.93
C18:2n6c	3–4	7.44 ± 0.64 ^ay^	4.14 ± 0.69 ^az^	13.71 ± 3.12 ^ax^
	6–7	3.89 ± 0.33 ^bxy^	2.98 ± 0.99 ^aby^	6.51 ± 2.66 ^bx^
	9–10	4.80 ± 2.02 ^bxy^	2.66 ± 0.44 ^by^	6.21 ± 3.29 ^bx^
	Effect	*P*_age_ < 0.05	*P*_muscle_ < 0.05	*P*_age×muscle_ < 0.05
C18:3n6	3–4	0.06 ± 0.01 ^axy^	0.04 ± 0.01 ^ay^	0.08 ± 0.03 ^ax^
	6–7	0.05 ± 0.01 ^ax^	0.04 ± 0.01 ^ax^	0.06 ± 0.02 ^ax^
	9–10	0.05 ± 0.01 ^axy^	0.04 ± 0.01 ^ay^	0.06 ± 0.01 ^ax^
	Effect	*P*_age_ < 0.05	*P*_muscle_ < 0.05	*P*_age×muscle_ = 0.48
C18:3n3	3–4	1.38 ± 0.18 ^ax^	1.38 ± 0.28 ^ax^	1.45 ± 0.34 ^ax^
	6–7	1.05 ± 0.34 ^ax^	0.82 ± 0.24 ^bx^	1.06 ± 0.18 ^bx^
	9–10	1.09 ± 0.18 ^ax^	0.83 ± 0.10 ^by^	1.02 ± 0.21 ^bxy^
	Effect	*P*_age_ < 0.05	*P*_muscle_ = 0.13	*P*_age×muscle_ = 0.76
C20:2	3–4	0.08 ± 0.01 ^ay^	0.05 ± 0.01 ^az^	0.12 ± 0.03 ^ax^
	6–7	0.03 ± 0.01 ^bx^	0.03 ± 0.01 ^ax^	0.05 ± 0.02 ^bx^
	9–10	0.05 ± 0.03 ^abx^	0.03 ± 0.01 ^ax^	0.05 ± 0.03 ^bx^
	Effect	*P*_age_ < 0.05	*P*_muscle_ < 0.05	*P*_age×muscle_ = 0.06
C20:3n6	3–4	0.23 ± 0.03 ^ay^	0.09 ± 0.03 ^az^	0.46 ± 1.38 ^ax^
	6–7	0.11 ± 0.01 ^bxy^	0.08 ± 0.04 ^ay^	0.24 ± 0.13 ^bx^
	9–10	0.12 ± 0.05 ^bxy^	0.06 ± 0.11 ^ay^	0.18 ± 0.11 ^bx^
	Effect	*P*_age_ < 0.05	*P*_muscle_ < 0.05	*P*_age×muscle_ < 0.05
C20:3n3	3–4	0.05 ± 0.01 ^ax^	0.05 ± 0.01 ^ax^	0.04 ± 0.02 ^ax^
	6–7	0.04 ± 0.01 ^ax^	0.03 ± 0.01 ^bx^	0.04 ± 0.00 ^ax^
	9–10	0.38 ± 0.01 ^ax^	0.03 ± 0.00 ^bx^	0.04 ± 0.02 ^ax^
	Effect	*P*_age_ < 0.05	*P*_muscle_ = 0.68	*P*_age×muscle_ = 0.36
C20:4	3–4	0.03 ± 0.01 ^ay^	0.01 ± 0.01 ^ay^	0.06 ± 0.02 ^ax^
	6–7	0.01 ± 0.00 ^bx^	0.01 ± 0.00 ^ax^	0.02 ± 0.01 ^bx^
	9–10	0.01 ± 0.00 ^bx^	0.01 ± 0.00 ^ax^	0.01 ± 0.01 ^bx^
	Effect	*P*_age_ < 0.05	*P*_muscle_ < 0.05	*P*_age×muscle_ < 0.05
C22:2n6	3–4	0.05 ± 0.02 ^ax^	0.03 ± 0.01 ^ax^	0.05 ± 0.03 ^ax^
	6–7	0.04 ± 0.02 ^ax^	0.03 ± 0.01 ^ax^	0.04 ± 0.01 ^ax^
	9–10	0.04 ± 0.13 ^ax^	0.26 ± 0.08 ^ax^	0.04 ± 0.02 ^ax^
	Effect	*P*_age_ = 0.15	*P*_muscle_ < 0.05	*P*_age×muscle_ = 0.92
C20:5n3	3–4	0.46 ± 0.08 ^ax^	0.15 ± 0.03 ^ay^	0.67 ± 0.31 ^ax^
	6–7	0.27 ± 0.03 ^bxy^	0.19 ± 0.13 ^ay^	0.53 ± 0.26 ^ax^
	9–10	0.27 ± 0.09 ^bxy^	0.11 ± 0.03 ^ay^	0.38 ± 0.18 ^ax^
	Effect	*P*_age_ < 0.05	*P*_muscle_ < 0.05	*P*_age×muscle_ = 0.36
C22:6n3	3–4	0.08 ± 0.01 ^ax^	0.03 ± 0.01 ^ax^	0.09 ± 0.08 ^ax^
	6–7	0.07 ± 0.04 ^ax^	0.04 ± 0.02 ^ax^	0.09 ± 0.05 ^ax^
	9–10	0.05 ± 0.03 ^ax^	0.04 ± 0.00 ^ax^	0.11 ± 0.06 ^ax^
	Effect	*P*_age_ = 0.98	*P*_muscle_ < 0.05	*P*_age×muscle_ = 0.83
SFA	3–4	55.76 ± 1.55 ^ay^	58.90 ± 2.08 ^ax^	47.17 ± 1.89 ^az^
	6–7	59.58 ± 1.56 ^ax^	57.96 ± 5.41 ^axy^	51.53 ± 5.65 ^ay^
	9–10	58.15 ± 3.97 ^axy^	59.77 ± 2.97 ^ax^	51.42 ± 7.57 ^ay^
	Effect	*P*_age_ = 0.20	*P*_muscle_ < 0.05	*P*_age×muscle_ = 0.63
UFA	3–4	44.23 ± 4.53 ^ay^	41.10 ± 2.07 ^az^	52.83 ± 1.90 ^ax^
	6–7	40.42 ± 1.56 ^ay^	42.03 ± 5.41 ^axy^	48.47 ± 5.66 ^ax^
	9–10	41.85 ± 3.97 ^axy^	40.23 ± 2.97 ^ay^	48.58 ± 7.57 ^ax^
	Effect	*P*_age_ = 0.20	*P*_muscle_ < 0.05	*P*_age×muscle_ = 0.64
MUFA	3–4	34.30 ± 1.51 ^ax^	35.09 ± 1.86 ^ax^	36.09 ± 3.85 ^ax^
	6–7	34.81 ± 1.03 ^ay^	37.74 ± 4.17 ^axy^	39.86 ± 2.68 ^ax^
	9–10	35.26 ± 2.01 ^ax^	36.37 ± 2.87 ^ax^	40.48 ± 5.25 ^ax^
	Effect	*P*_age_ = 0.09	*P*_muscle_ < 0.05	*P*_age×muscle_ = 0.65
PUFA	3–4	9.94 ± 0.89 ^ay^	6.02 ± 0.90 ^az^	16.75 ± 3.95 ^ax^
	6–7	5.61 ± 0.59 ^by^	4.30 ± 1.32 ^bxy^	8.61 ± 3.14 ^bx^
	9–10	6.59 ± 2.29 ^bxy^	3.85 ± 0.44 ^by^	8.11 ± 3.76 ^bx^
	Effect	*P*_age_ < 0.05	*P*_muscle_ < 0.05	*P*_age×muscle_ < 0.05
UFA/SFA	3–4	0.18 ± 0.19 ^ay^	0.10 ± 0.01 ^ay^	0.36 ± 0.09 ^ax^
	6–7	0.10 ± 0.01 ^by^	0.08 ± 0.03 ^ay^	0.17 ± 0.07 ^bx^
	9–10	0.12 ± 0.48 ^bxy^	0.07 ± 0.01 ^ay^	0.17 ± 0.09 ^bx^
	Effect	*P*_age_ < 0.05	*P*_muscle_ < 0.05	*P*_age×muscle_ < 0.05
n-6/n-3 PUFA	3–4	3.93 ± 0.29 ^ay^	2.71 ± 0.47 ^az^	6.30 ± 0.87 ^ax^
	6–7	2.98 ± 0.70 ^ax^	2.94 ± 0.54 ^ax^	3.89 ± 1.17 ^bx^
	9–10	3.33 ± 0.98 ^ax^	2.79 ± 0.65 ^ax^	3.98 ± 1.48 ^bx^
	Effect	*P*_age_ < 0.05	*P*_muscle_ < 0.05	*P*_age×muscle_ < 0.05
n-6 PUFA	3–4	7.53 ± 0.66 ^ay^	4.18 ± 0.69 ^az^	13.77 ± 3.13 ^ax^
	6–7	3.95 ± 0.32 ^bxy^	3.03 ± 0.99 ^aby^	6.55 ± 2.66 ^bx^
	9–10	4.87 ± 2.01 ^bxy^	2.69 ± 0.45 ^by^	6.24 ± 3.27 ^bx^
	Effect	*P*_age_ < 0.05	*P*_muscle_ < 0.05	*P*_age×muscle_ < 0.05
n-3 PUFA	3–4	1.92 ± 0.21 ^ax^	1.57 ± 0.31 ^ax^	2.00 ± 0.65 ^ax^
	6–7	1.39 ± 0.35 ^bxy^	1.05 ± 0.33 ^by^	1.63 ± 0.38 ^ax^
	9–10	1.42 ± 0.22 ^bx^	0.98 ± 0.12 ^by^	1.50 ± 0.39 ^ax^
	Effect	*P*_age_ < 0.05	*P*_muscle_ < 0.05	*P*_age×muscle_ = 0.97

^a–c^ The variables with the same letters in the same column do not differ (*p* ≥ 0.05). ^x–z^ The variables with the same letters in the same row do not differ (*p* ≥ 0.05). *P*_age_, *P*_muscle_, and *P*_age×muscle_ are the effects of age, muscle type, and the age × muscle type interaction on the fatty acids of meat, respectively. ND stands for undetected; The unit of the fatty acid content was %, and the ratio unit was 1. SFA: saturated fatty acid; MUFA: mono-unsaturated fatty acids; PUFA: poly-unsaturated fatty acids; SFA: total saturated fatty acids; UFA: total unsaturated fatty acids. Butyric acid (C4:0); caproic acid (C6:0); caprylic acid (C8:0); capric acid (C10:0); undecanoic acid (C11:0); lauric acid (C12:0); tridecanoic acid (C13:0); myristic acid (C14:0); myristoleic acid (C14:1); pentadecanoic acid (C15:0); cis-10-Pentadecenoic acid (C15:1); palmitic acid (C16:0); palmitoleic acid (C16:1); heptadecanoic acid (C17:0); cis-10-heptadecenoic acid (C17:1); stearic acid (C18:0); oleic acid (C18:1n9c); elaidic acid (C18:1n9t); linoleic acid (C18:2n6c); linolelaidic acid (C18:2n6t); γ-linolenic acid (C18:3n6); α-linolenic acid (C18:3n3); arachidic acid (C20:0); cis-11-eicosenoic acid (C20:1); cis-11,14-eicosadienoic acid (C20:2); cis-8,11,14-eicosatrienoic acid (C20:3n6); cis-11,14,17-eicosatrienoic acid (C20:3n3); arachidonic acid (C20:4n6); cis-5,8,11,14,17-eicosapentaenoic acid (C20:5n3); heneicosanoic acid (C21:0); behenic acid (C22:0); erucic acid (C22:1); cis-13,16-docosadienoic acid (C22:2n6); cis-4,7,10,13,16,19-docosahexaenoic acid (C22:6n3); tricosanoic acid (C23:0); lignoceric acid (C24:0); nervonic acid (C24:1).

**Table 5:** Means and standard errors for meat quality characteristics of the *Psoas major* (PM), *Longissimus thoracic* (LT), and *semitendinosus* (ST) muscle samples from Alxa Bactrian camels (*n* = 5, mean ± standard error) slaughtered at three different ages.

Measurement	Age (Year)	Muscle		
		PM	LT	ST
Ultimate pH	3–4	5.55 ± 0.12 ^ay^	6.08 ± 0.29 ^ax^	6.12 ± 0.33 ^ax^
	6–7	5.52 ± 0.15 ^ay^	5.75 ± 0.12 ^abx^	5.67 ± 0.11 ^bx^
	9–10	5.61 ± 0.18 ^ax^	5.49 ± 0.10 ^bx^	5.84 ± 0.34 ^abx^
	Effect	*P*_age_ < 0.05	*P*_muscle_ < 0.05	*P*_age×muscle_ < 0.05
Cooking loss%	3–4	30.90 ± 1.27 ^ax^	28.93 ± 1.24 ^ay^	27.64 ± 0.62 ^ay^
	6–7	28.83 ± 0.70 ^bx^	27.66 ± 1.10 ^abxy^	26.76 ± 0.65 ^ay^
	9–10	27.51 ± 1.40 ^bx^	26.53 ± 1.31 ^bx^	24.64 ± 0.81 ^by^
	Effect	*P*_age_ < 0.05	*P*_muscle_ < 0.05	*P*_age×muscle_ = 0.64
Shear value (N)	3–4	84.57 ± 3.32 ^cy^	80.74 ± 1.65 ^cy^	93.52 ± 2.53 ^cx^
	6–7	102.51 ± 6.64 ^by^	93.66 ± 2.81 ^by^	112.36 ± 7.88 ^bx^
	9–10	121.27 ± 6.29 ^ay^	115.80 ± 4.17 ^ay^	132.49 ± 4.95 ^ax^
	Effect	*P*_age_ < 0.05	*P*_muscle_ < 0.05	*P*_age×muscle_ = 0.67
Color parameters				
L* (lightness)	3–4	39.78 ± 1.02 ^ax^	38.77 ± 1.87 ^axy^	36.75 ± 2.28 ^ay^
	6–7	37.04 ± 1.45 ^bxy^	38.06 ± 2.01 ^ax^	35.34 ± 3.25 ^aby^
	9–10	36.14 ± 1.73 ^bxy^	37.61 ± 3.05 ^ax^	34.72 ± 2.79 ^by^
	Effect	*P*_age_ < 0.05	*P*_muscle_ < 0.05	*P*_age×muscle_ = 0.82
a* (redness)	3–4	17.13 ± 0.88 ^bx^	17.74 ± 1.91 ^bx^	17.29 ± 0.99 ^ax^
	6–7	19.31 ± 1.06 ^abx^	18.92 ± 3.79 ^bx^	17.36 ± 1.56 ^ax^
	9–10	20.01 ± 2.43 ^axy^	23.08 ± 0.99 ^ax^	17.99 ± 1.72 ^ay^
	Effect	*P*_age_ < 0.05	*P*_muscle_ < 0.05	*P*_age×muscle_ = 0.16
b* (yellowness)	3–4	8.12 ± 0.47 ^bx^	9.71 ± 2.68 ^ax^	8.32 ± 1.25 ^ax^
	6–7	9.22 ± 0.73 ^bxy^	10.10 ± 1.72 ^ax^	7.40 ± 0.82 ^ay^
	9–10	10.62 ± 0.98 ^ax^	10.59 ± 1.07 ^ax^	8.42 ± 1.43 ^ax^
	Effect	*P*_age_= 0.13	*P*_muscle_ < 0.05	*P*_age×muscle_ = 0.44

^a–c^ The variables with the same letters in the same column do not differ (*p* ≥ 0.05). ^x–z^ The variables with the same letters in the same row do not differ (*p* ≥ 0.05). *P*_age_, *P*_muscle_, and *P*_age×muscle_ The effects of age, muscle type, and the age × muscle type interaction on quality characteristics of meat, respectively.

## Data Availability

Data are contained within the article.
